# Current management of chronic kidney disease in type‐2 diabetes—A tiered approach: An overview of the joint Association of British Clinical Diabetologists and UK Kidney Association (ABCD‐UKKA) guidelines

**DOI:** 10.1111/dme.15450

**Published:** 2024-10-17

**Authors:** Indranil Dasgupta, Sagen Zac‐Varghese, Khuram Chaudhry, Kieran McCafferty, Peter Winocour, Tahseen A. Chowdhury, Srikanth Bellary, Gabrielle Goldet, Mona Wahba, Parijat De, Andrew H. Frankel, Rosa M. Montero, Eirini Lioudaki, Debasish Banerjee, Ritwika Mallik, Adnan Sharif, Naresh Kanumilli, Nicola Milne, Dipesh C. Patel, Ketan Dhatariya, Stephen C. Bain, Janaka Karalliedde

**Affiliations:** ^1^ Heartlands Hospital, Birmingham and Warwick Medical School University of Warwick Coventry UK; ^2^ East and North Herts NHS Trust Hertfordshire UK; ^3^ Guy's and St Thomas' Hospital London UK; ^4^ Barts Health NHS Trust London UK; ^5^ Royal London Hospital London UK; ^6^ Aston University Birmingham UK; ^7^ Imperial College Healthcare NHS Trust London UK; ^8^ Epsom & St Helier University NHS Trust London UK; ^9^ City Hospital Birmingham UK; ^10^ St George's Hospital London UK; ^11^ King's College Hospital London UK; ^12^ St George's University Hospitals NHS Foundation Trust London UK; ^13^ University College London NHS Trust London UK; ^14^ University Hospitals Birmingham Birmingham UK; ^15^ Northenden Group Practice Manchester UK; ^16^ Greater Manchester Diabetes Clinical Network Manchester UK; ^17^ Royal Free London NHS Foundation Trust London UK; ^18^ Norfolk and Norwich University Hospitals NHS Foundation Trust and Norwich Medical School University of East Anglia Norwich UK; ^19^ Swansea University Swansea UK; ^20^ Guy's and St Thomas' Hospital London and King's College London London UK

**Keywords:** ACE inhibitior, hypertension, kidney disease, lifestyle, type 2 diabetes

## Abstract

A growing and significant number of people with diabetes develop chronic kidney disease (CKD). Diabetes‐related CKD is a leading cause of end‐stage kidney disease (ESKD) and people with diabetes and CKD have high morbidity and mortality, predominantly related to cardiovascular disease (CVD). Despite advances in care over the recent decades, most people with CKD and type 2 diabetes are likely to die of CVD before developing ESKD. Hyperglycaemia and hypertension are modifiable risk factors to prevent onset and progression of CKD and related CVD. People with type 2 diabetes often have dyslipidaemia and CKD per se is an independent risk factor for CVD, therefore people with CKD and type 2 diabetes require intensive lipid lowering to reduce burden of CVD. Recent clinical trials of people with type 2 diabetes and CKD have demonstrated a reduction in composite kidney end point events (significant decline in kidney function, need for kidney replacement therapy and kidney death) with sodium‐glucose co‐transporter‐2 (SGLT‐2) inhibitors, non‐steroidal mineralocorticoid receptor antagonist finerenone and glucagon‐like peptide 1 receptor agonists. The Association of British Clinical Diabetologists (ABCD) and UK Kidney Association (UKKA) Diabetic Kidney Disease Clinical Speciality Group have previously undertaken a narrative review and critical appraisal of the available evidence to inform clinical practice guidelines for the management of hyperglycaemia, hyperlipidaemia and hypertension in adults with type 2 diabetes and CKD. This 2024 abbreviated updated guidance summarises the recommendations and the implications for clinical practice for healthcare professionals who treat people with diabetes and CKD in primary, community and secondary care settings.

**What’s new?**

- Diabetes is the commonest cause of CKD and ESKD globally and these people have a very high risk of CVD and death.
- Trial data confirm that the rate of progression to ESKD, CVD and mortality in people with with CKD due to type‐2 diabetes can be can be effectively mitigated by established and newer interventions.
- Recent studies demonstrate stark inadequacies and inequities in the management of diabetic CKD in the UK and elsewhere.
- The updated ABCD‐UKKA Guidlines incorporate all recent trial evidences and propose a tiered approach to implementation of the recommendations, to promote a holistic, individualised mutiple‐risk factor intervention.

## 1 INTRODUCTION

Diabetes is the commonest cause of chronic kidney disease (CKD) globally accounting for 42% of cases.^1^ In the United Kingdom (UK), half of the cases of CKD and one‐third of end‐stage kidney disease (ESKD) starting kidney replacement therapy (KRT) are attributed to diabetes.^1^, ^2^ Type 2 diabetes (T2D) is one the fastest growing health challenges of the twenty‐first century globally and contributes to high economic costs of diabetes, which in the UK National Health Service (NHS) is approximately £10 billion per year. Of these costs 80% is spent on treating complications of diabetes such as CKD, placing a significant demand on health and social care services.^3^ Currently, there are five million people with diabetes in the UK, 40% of whom will develop CKD in their lifetime and many of these people will be from disadvantaged communities and groups with evidence of health inequality and inequity with regard to kidney disease.^3^, ^4^, ^5^

CKD is associated with a very high risk of cardiovascular disease (CVD), which increases steeply with the progression of CKD.^4^, ^6^ Indeed, most people with CKD are likely to die of CVD rather than need kidney replacement therapy (KRT).^7^ Currently, in the UK, CKD accounts for 45,000 premature deaths and over 100,000 hospital admissions a year, mainly for cardiovascular events.^4^

Early interventions that include lifestyle modification, good glycaemic and blood pressure (BP) control, and the use of renin‐angiotensin‐aldosterone system inhibitors (RASi) and statins can slow the progression of CKD and reduce CVD risk.^7^ In the past few years, evidence has emerged for newer interventions; sodium‐glucose co‐transporter‐2 inhibitors (SGLT2i), non‐steroidal selective mineralocorticoid receptor antagonist (nsMRA) finerenone, and glucagon‐like peptide 1 receptor agonist (GLP1 RA) that can further reduce the risk of ESKD and CVD (including heart failure) in people with CKD.^8^, ^9^, ^10^

With the advent of the newer interventions, the Joint Association of British Clinical Diabetologists and UK Kidney Association (ABCD‐UKKA) Committee have updated the guidelines on glycaemic, BP and lipid management and produced a consensus statement on the use of finerenone in progressive CKD.^11^, ^12^, ^13^, ^14^ This article is an overview of these four documents focussing on a practical, individualised approach to applying the recommendations to improve cardio‐renal outcome in people with type 2 diabetes and CKD which is an imperative given the enormous human and societal impact of CKD in this cohort.

## 2 MANAGEMENT OF CKD: A TIERED APPROACH

**FIGURE 1:**
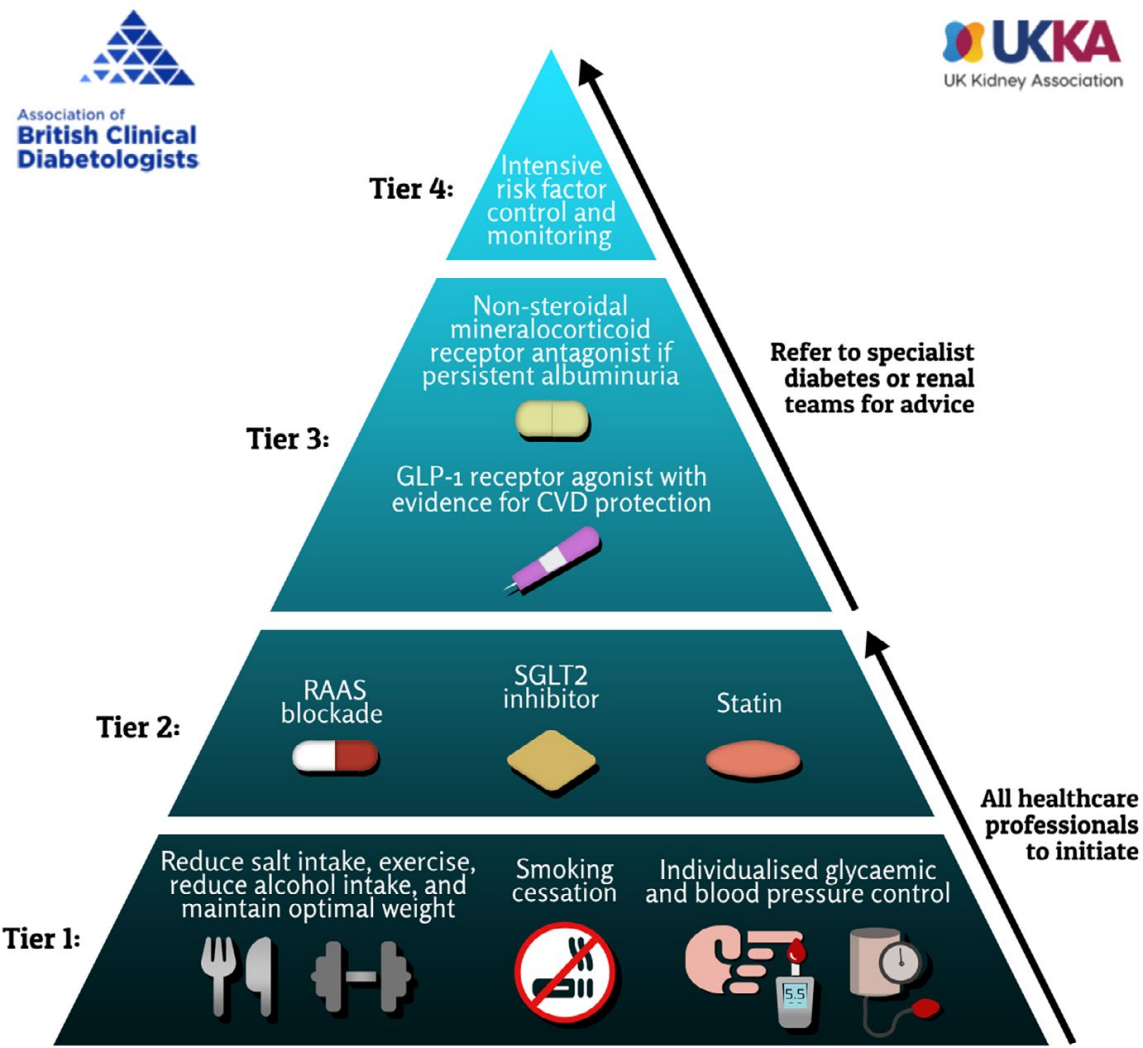
Management of CKD in type‐2 diabetes: a tiered approach.

The pathogenesis of CKD involves a complex interplay of multiple mechanisms including haemodynamic, metabolic and inflammatory processes leading to progressive kidney damage and fibrosis.^15^ Therefore, multiple risk factor interventions are necessary to stem the progression of CKD. The ABCD‐UKKA guidelines recommend multicomponent risk factor interventions based on the current evidence. The benefit of multi‐component intervention in T2D and CKD is in reducing the risk of ESKD, CVD and death is well established.^16^, ^17^, ^18^ However, implementation of even the long‐established interventions is inadequate in routine practice, leading to significant unmet needs and inequity in the management of T2D and CKD in the UK and many countries worldwide.^19^, ^20^ We believe the tiered approach will help to address the unmet needs and inequity and suggest that Tiers 1 and 2 management should be instituted in primary care, while Tiers 3 and 4 management require collaboration between primary and secondary care based on the relative risks of kidney disease progression and CVD (Figure 1).

Young people with diabetes and CKD have a substantially elevated lifetime cardiovascular risk compared to those without CKD. There are few intervention trials in these groups and the significantly elevated cardiovascular risk would be the justification for treatment. Within the ABCD‐UKKA guidelines, we have suggested an individualised approach to monitoring and treatment in this group with consideration of additional cardiovascular risk factors and high lifetime cardio‐renal risk which would warrant intensive treatment.

Women of childbearing potential with CKD and diabetes need to be counselled about increased risks of pregnancy complications both to the mother and to the unborn baby, and the need to stop potentially teratogenic or unlicensed treatments during pre‐conception and pregnancy. A comprehensive pre‐conception medical review and assessment of the safety of all medications as well counselling on CKD‐associated risk is required.^21^ It is important for all clinicians to be aware of and advise on contraception in women of childbearing age when on potentially teratogenic or unlicensed treatments in pregnancy.

The evidence for intensive BP, glucose and lipid management in frail people living with diabetes and CKD is scarce, and an individualised approach is recommended for initiation, monitoring and cessation of therapy in those cohort.^22^

### 2.1 Tier 1

The basic building blocks for the management of type 2 diabetes and CKD are lifestyle changes, individualised glycaemic and BP management. These have been part of the standard of care in all the clinical trials demonstrating the benefit of various therapeutic interventions in reducing kidney and CVD outcomes in people with type 2 diabetes and CKD.^7^, ^8^, ^9^, ^10^

#### 2.1.1 Lifestyle changes

The five main elements of lifestyle advice are reduced salt intake <90 mmol of sodium daily (<2 g of sodium or <5 g of sodium chloride daily), alcohol <14 units a week, smoking cessation, regular exercise at least 30 min daily for 5 days a week, and to maintain a body mass index between 20 and 25 kg/m^2^.^12^ Individually and collectively, they have been shown to improve glycaemic and BP control, and CVD risk, although the evidence is mainly observational. It is recommended that the advice is reinforced on a regular basis.

#### 2.1.2 Glycaemic management

The management of hyperglycaemia in diabetes is predicated on the basis of reducing hyperglycaemia to improve osmotic symptoms, with supportive evidence that this will prevent the onset, and slow down progression, of kidney and vascular complications over time.^10^

The guideline focusses on the need for an individualised approach to care and targets for glucose control. It is important to highlight the lack of studies of high‐quality evidence in the context of intensive glucose control per se improving outcomes in people with established CKD.

Moreover, the greater risk for hypoglycaemia in CKD is acknowledged especially with the use of insulin or sulfonylureas/glinides as kidney function deteriorates. Hence individualised, pragmatic glycaemic goals that balance the benefits and risks of intensive glucose lowering in people with diabetes and CKD, and patient education on hypoglycaemia avoidance and self‐management are needed. There has also been an important shift in emphasis with recent guidance for people with type 2 diabetes at high‐risk for adverse cardio‐renal outcomes. Specific emphasis should be placed on selecting medications for their cardio‐renal benefits independent of their glucose‐lowering effects for this high‐risk cohort.^11^

Metformin has been used as a first‐line oral drug for people with type 2 diabetes for over 60 years. The dose of metformin should be decreased to 500 mg twice a day if eGFR is less than 45 mL/min/1.73 m^2^ and omitted in the majority of circumstances if eGFR is less than 30 mL/min/1.73 m^2^. Of note limitations of eGFR measurements need to be appreciated especially in those at extremes of body weight and individualised treatment decisions may need to be considered in people with eGFR 25–30 mL/min/1.73m^2^.^11^

The guidelines acknowledge that there is a lack of high‐quality clinical trial evidence for the use of metformin as a cardio‐renal protective agent in people with CKD but in most countries worldwide it remains the most widely used first‐line agent for type 2 diabetes.

Three large clinical trials have consistently shown that SGLT‐2 inhibition on top of standard of care (RAS inhibition) significantly reduces the risk of progression of CKD in people with type 2 diabetes and CKD.^10^ The observed kidney and cardiovascular benefits of SGLT‐2 inhibitors are independent of the HbA1c‐lowering effects of these agents in people with type 2 diabetes. In people with diabetes and eGFR<45 mL/min/1.73 m2, treatment with SGLT‐2 inhibitors does not lower HbA1c significantly. SGLT‐2 inhibitors can be initiated for kidney protection above an eGFR >20 mL/min/1.73m^2^, however, if the GFR is below 45 and further glucose lowering is required adding another class of medications to optimise diabetes control is recommended.

Systematic reviews and meta‐analyses suggest a clear beneficial class effect GLP‐1RA on the CVD risk and albuminuria reduction.^23^, ^24^ Currently, there is one primary kidney end point study showing cardio‐renal benefits of semaglutide compared to placebo^9^ on top of standard of care (RASi). Of note ~15.6% of the cohort were on SGLT‐2 inhibitors at baseline and no major heterogeneity of treatment effect was observed in those on this combination. The available data to date from secondary or sub‐analyses of trials, simulation‐type analyses from recent studies and real‐world data suggest potential complementary and additive effects and in our opinion combination treatment GLP‐1 RA and SGLT‐2 inhibitors should be considered early in the management of CKD in people with diabetes.^10^ Such a combination approach may aid cardio‐renal risk reduction by targeting residual risk and also improve glycaemic control with low risk of hypoglycaemia in people with type 2 diabetes and CKD.^25^

#### 2.1.3 Hypertension management

The guideline emphasises the importance of accurate BP measurement for the diagnosis of hypertension and monitoring of BP after starting treatment and suggests using standardised BP measurement technique as per the British and Irish Hypertension Society (BIHS) guidance.^26^ The mean difference in systolic BP between standardised and routine measurements is around 13 mmHg with wide limits of agreement (−46.1 to 20.7 mm Hg).^27^ The suggested threshold for starting pharmacological treatment varies depending on the type of diabetes, the age of the person, presence or absence of CKD and albuminuria.^26^

BP control is the most important measure to slow progression of CKD in people with diabetes. For those with urine albumin creatinine ratio (ACR) 3‐30 mg/mmol, CKD stages 1–3 or stages 4–5 with ACR >30 mg/mmol, the guidelines recommend a target BP of ≤130/80 mmHg. For those with CKD stages 4–5 without albuminuria or who are on dialysis, the recommended target is ≤140/90 mmHg.^26^

Angiotensin‐converting enzyme (ACE) inhibitors are the suggested first‐line anti‐hypertensive agents for CKD. If ACE inhibitors are not tolerated, angiotensin receptor blockers (ARBs) should be prescribed. If BP is not controlled to target, we suggest following the National Institute for Health and Care Excellence (NICE) treatment algorithm to select the second line and subsequent anti‐hypertensive agents.^28^ In people with advanced CKD and treatment‐resistant hypertension (uncontrolled hypertension despite treatment with three or more agents at maximum tolerated doses including a diuretic), adding a thiazide diuretic may improve BP control.^29^

### 2.2 Tier 2

Although referred to as Tier 2, the interventions in this tier may be started concurrently with the interventions in Tier 1. The main objective here is to reduce the baseline risks of CKD progression and CVD. Both Tiers 1 and 2 management should be started in primary care.

#### 2.2.1 Use of renin‐angiotensin blocking agents

Over and above the BP lowering effect, RASi, ACE inhibitor or ARBs further reduce the risk of progression of CKD. In the landmark RENAAL and IDNT trials, there was 16%–20% reduction in the risk of kidney disease progression, but a significant number of people studied had residual risk despite RASi.^25^, ^30^, ^31^ Furthermore, no significant reduction in CVD events was demonstrated in these trials. For maximum kidney protection, the dose of ACE inhibitor or ARB should be titrated up to the maximum tolerated, keeping an eye on serum potassium and creatinine.

Serum electrolytes and creatinine (eGFR) should be measured before starting and 1–2 week after starting or increasing the dose of RASi. A reduction in eGFR is anticipated after starting or increasing the dose of RASi. If the eGFR falls >25%, stop the drug, repeat the test, and consider other causes, for example, volume depletion, non‐steroidal anti‐inflammatory use. If the reduction in eGFR is <25%, we suggest continuing the drug and repeating the test in further 1–2 weeks. Similarly, a rise in serum potassium is expected with starting and increasing the dose of RASi. We suggest continuing the drug if the serum potassium is <6.0 mmol/L, with dietary advice for lowering potassium containing food, and repeat the test in 1–2 week.

Dual RAS inhibition has not been shown to provide additional benefit and in fact may be detrimental in terms of risk of hyperkalaemia and deterioration in kidney function.^32^ As such, combining ACE inhibitor and ARB is discouraged. It is recommended that RAS blocking agents are temporarily stopped during periods of acute illness to prevent acute kidney injury. The STOP ACE trial has demonstrated that stopping RAS blocking agents in progressive CKD does not improve kidney outcomes and hence this class of agent should be continued in people with more advanced progressive CKD (eGFR, <30 mL per min per 1.73 m^2^.).^33^

#### 2.2.2 Use of SGLT‐2 inhibitors

Although RAS blockade slows progression of CKD, in the trials around 40% of patients still met the primary kidney end points. The recent SGLT2 inhibitor trials have shown further reduction in the risk of progression of kidney disease by around 30%. These trials have also demonstrated reduction in risk of CVD and mortality. Furthermore, patients taking SGLT2i in the trial had on an average 3 mmHg lower systolic BP which may also help to protect the kidney. The trials had different inclusion and exclusion criteria, but all showed significant renal benefit and thus no one agent can be recommended above the other. We recommend using SGLT2i in all patients with CKD in T2D eGFR >15 mL/min/1.73m^2^.

#### 2.2.3 Lipid‐lowering therapy

The ABCD‐UKKA guidelines recommend most people with type 2 diabetes and CKD should receive an optimum dose of a statin for primary prevention.^13^ Those, who do not tolerate a statin, should receive ezetimibe either alone or with a small dose of a statin if tolerated. Further management of dyslipidaemia is detailed in the guideline.^13^ Large studies have shown that a 1 mmol/L reduction in low‐density lipoprotein (LDL) cholesterol reduces cardiovascular events by 21%.^34^ These benefits are seen in people with diabetes and in CKD. However, smaller effects are evident as eGFR declines with no benefit seen in people on KRT with haemodialysis.^35^ However, following kidney transplantation, lipid lowering reduces major adverse cardiovascular events.^36^ Meta‐analyses have demonstrated that larger reductions in LDL cholesterol led to further reductions in major vascular events and indeed there is no evidence of adverse effects with more intensive LDL lowering treatment.^37^

For people with type 2 diabetes and CKD, we suggest the following treatment targets: total cholesterol ≤4.0 mmol/L, non‐HDL cholesterol ≤2.5 mmol/L and LDL cholesterol ≤1.8 mmol/L.^12^ For pragmatic reasons we have not suggested percentage reductions in baseline levels, nor have we suggested a graded approach to therapy. We are aware that with time pressures and conflicting demands, lipid management is often neglected. Clear targets and a simple stepwise approach to management can help more people access appropriate therapy.

In most cases, atorvastatin 20 mg is suggested as the first‐line lipid‐lowering agent with dose titration or use of higher intensity statins as required and according to summary of product characteristics (SPC) and local guidance. Specialist advice should be sought at eGFR <30 mL/min/1.73 m^2^. In people with statin intolerance, ezetimibe alone or in combination with bempedoic acid can be used. Fibrates cause a reversible increase in creatinine and specialist advice is recommended when these are used in people with CKD.^13^

There are newer agents, inclisiran, PSCK9 inhibitors and icosapent ethyl, available for people who do not meet treatment targets using statins or ezetimibe. These agents have specific licensing indications (including specific cholesterol ranges) and also local restrictions. For these agents, evidence for benefit exists up to stage G3b CKD.^13^

Lipid‐lowering therapy is effective in lowering CVD risk, however, there are specific side effects to be aware of which are listed on the individual summary of product characteristics, and these should be appropriately discussed prior to medication initiation.

### 2.3 Tier 3

In the SGLT2i trials, the use of SGLT2 inhibitors over and above the standard of care treatment, which included BP and glycaemic control and optimum RASi, around 10% of patients reached primary kidney end points and around 7% reached CVD end points.^25^, ^38^ This suggests despite optimum Tier 2 treatment there remains residual risk of kidney disease progression and CVD in a significant number of patients, for whom we suggest the following interventions.

#### 2.3.1 Use of finerenone

Mineralocorticoid receptor antagonists (MRAs) like spironolactone and eplerenone have been demonstrated to improve blood pressure control, reduce proteinuria and slow progression of CKD and reduce mortality in heart failure.^39^, ^40^, ^41^ However, their use is limited by their propensity to cause hyperkalaemia in people with CKD, especially those with diabetes. Finerenone is a non‐steroidal, selective MRA with greater mineralocorticoid receptor affinity and selectivity, and as such its use is associated with less hyperkalaemia and minimal gynaecomastia compared with the steroidal MRAs.^42^

Based on the strong evidence of cardio‐renal protection offered by the addition of finerenone in the FIDELIO‐CKD and FIGARO‐CKD studies,^43^ we suggest in people with CKD who have persistent albuminuria (ACR >30 mg/mmol) despite the use of maximum tolerated doses of RASi and SGLT2i, to consider addition of finerenone to reduce the risk of adverse kidney and cardiovascular outcomes. Finerenone can be used if eGFR is more than or equal to 25 mL/min/1.73m^2^ and serum potassium is normal (<5 mmol/L). Finerenone can also be used as a second line drug in addition to ACE inhibitor or ARB if SGLT2i is not tolerated. It is important to monitor serum potassium level after commencing treatment and the dose of finerenone adjusted accordingly.^14^

#### 2.3.2 Use of GLP‐1 receptor agonists (GLP‐1 RA)

At present, one primary kidney end point study reported with GLP‐1 RA^9^ which demonstrated kidney benefits and CVD mortality benefits with the use of semaglutide on top of RASi as compared to standard of care (RASi‐only). In this trial, ~15.6% of the cohort were on SGLT‐2 inhibitors at baseline and no major heterogeneity of treatment effect or results were observed in those on this combination. GLP‐1 RA also have robust data in people with type 2 diabetes with multiple clinical trials demonstrating CVD benefits in those at risk of CVD or with established CVD.^44^ The available limited data from recent studies and real‐world data suggest potential complementary and additive effects and in our opinion combination treatment GLP‐1 RA and SGLT‐2 inhibitors should also be considered early in the management people with T2D and CKD to address residual cardio‐renal risk (Tiers 1 or 2).^10^, ^25^, ^45^

### 2.4 Tier 4

For people with type 2 diabetes and CKD, CVD risk competes with the risk of progression to ESKD. Indeed, most people with CKD die of CVD before needing KRT. This is particularly true for older people with CKD as already mentioned. A critical assessment of peoples' relative risk of CVD and kidney failure should inform further intensification of treatment. Kidney Failure Risk Equation (KFRE, UK version) and QRISK3 are established risk calculators for CKD progression and CVD risks which we suggest are used in routine practice in the UK.^46^, ^47^ One may also use ‘CKD Patch’ which is a CVD and mortality risk predictor specifically for those with CKD for more accurate risk estimation.^48^

If the person's QRISK3 is high with a low KFRE despite optimum Tier 3 management, BP control and lipid management may be tightened further following the algorithms suggested in the ABCD‐UKKA guidelines.^11^, ^12^, ^13^, ^14^ Conversely, in a young person with high risk of CKD progression and CVD risk despite optimum Tier 3 management, BP control should be tightened, the dose of RAS blocking drug maximised and GLP‐1 RA added to afford further protection to the kidney. These persons will require closer monitoring of drug side effects, drug interactions, symptoms, BP and blood tests including serum potassium and creatinine (eGFR).

## 3 PATIENT ENGAGEMENT AND PRIMARY‐SECONDARY CARE COLLABORATION

Throughout the management pathway, people with T2D and CKD and their carers should be involved in the decision‐making process. Most people with CKD and type 2 diabetes on insulin monitor their capillary glucose levels at home. Adding home BP monitoring improves patient engagement in their care, adherence to prescribed medication and BP control.^49^ As many people with early‐stage CKD are cared for in primary care, supporting primary care teams in implementing the key elements of the guidelines (lifestyle, BP and glycaemic control, and the use of RASi, SGLT2 inhibitors and statins) is crucial to improving the outcomes and reducing the healthcare burden of CKD in people living with type 2 diabetes. Recent primary care research reinforces this need for education on CKD to enable early detection and intervention with a multidisciplinary team working approach, support from specialist teams essential to aid this process and implement guidelines in clinical practice.^19^, ^50^

## 4 CONCLUSION

The ABCD‐UKKA guidelines promote a holistic, individualised, multiple risk factor intervention with a view to improving cardio‐renal outcome in people with type 2 diabetes and CKD. While with the advent of the newer therapeutic agents, a ‘pillared approach’ to manage CKD has been proposed by many,^7^, ^51^, ^52^ we believe a tiered approach is more appropriate as it incorporates and emphasises the importance of the fundamental building blocks of CKD management including lifestyle changes, and BP and glycaemic control, and also allows individualisation of drug therapy based on age, relative CVD and kidney failure risks, person's wishes and expectations.

Internationally, multiple professional societies have produced recommendations on the management of CKD in Diabetes over the past few years, which can create the appearance of inconsistency and diminish implementation. Although developed following completely different processes, our recommendations broadly align with the Kidney Disease Global Outcome and American Diabetic Association (KDIGO‐ADA) Consensus Statement.^53^ We believe our guidelines should be easy to implement and improve the care and outcome of people with CKD.

## CONFLICT OF INTEREST STATEMENT

S.B. reports receiving personal fees from Abbott, AstraZeneca, Boehringer Ingelheim, Eli Lilly, Merck Sharp & Dohme, Novo Nordisk, and Sanofi Aventis and being a shareholder in Glycosmedia. D.B. reports receiving speaker fees from CSLVifor, Bayer; honoraria for advisory board from Bayer; and research grant from AstraZeneca. I.D. reports receiving research grants from Baxter, Medtronic and Sanofi‐Genzyme, receiving honoraria for attending advisory board and speaker meetings from GlaxoSmithKline, AstraZeneca, Vifor, Medtronic and Sanofi‐Genzyme, and being the national lead for 3 GSK trials. P.D. reports receiving honoraria for advisory work and/or lecture fees from AstraZeneca, Boehringer Ingelheim, Eli Lilly, Merck Sharp & Dohme, Napp Pharmaceuticals, Novo Nordisk, and Sanofi. J.K. reports receiving research grants from AstraZeneca and Sanofi and receiving speaker fees and attending advisory boards from Boehringer Ingelheim, AstraZeneca, Sanofi, and Napp. K.M. reports receiving speaker fees and attending advisory board from Vifor, AstraZeneca, Bayer, Boehringer Ingelheim, Pharmacomsos, Napp, Vifor Fresenius and receiving a grant from AstraZeneca. P.W. reports receiving honoraria for delivering educational meetings and/or attending advisory boards for Abbott, AstraZeneca, Bayer, Boehringer Ingelheim, Eli Lilly, Merck Sharp & Dohme, Napp, Sanofi, Novo, and Vifor Pharmaceuticals. K.D. reports receiving honoraria, travel or fees for speaking or advisory boards from AstraZeneca, Novo Nordisk, Boehringer Ingelheim, Eli Lilly, Abbott Diabetes, Menarini, Sanofi Diabetes and Roche. NK reports receiving speaker fees and advisory board fees from AstraZeneca, Bohringer Ingelheim, Lilly, Sanofi, Menarini, Novartis, Daiichi Sankyo. NM reports receiving fees for educational session and events, and advisory boards from Abbot, AstraZeneca, Bayer, Boehringer Ingelheim, Lilly, Menarini, Novo Nordisk, Roche, Sanofi. All the other authors declared no competing interests.

## Data Availability

Data sharing not applicable to this article as no datasets were generated or analysed during the current study.
